# Diversity and Abundance of the Species of Arboreal Mammals in a Tropical Rainforest in Southeast Mexico

**DOI:** 10.1002/ece3.70812

**Published:** 2025-01-20

**Authors:** J. Vladimir Rojas‐Sánchez, Rosamond Ione Coates, Víctor Sánchez‐Cordero, Mario C. Lavariega, José J. Flores‐Martínez

**Affiliations:** ^1^ Pabellón Nacional de la Biodiversidad, Departamento de Zoología, Instituto de Biología Universidad Nacional Autónoma de México Ciudad de México Mexico; ^2^ Posgrado en Ciencias Biológicas Universidad Nacional Autónoma de México Ciudad de México Mexico; ^3^ Estación de Biología Tropical Los Tuxtlas, Instituto de Biología Universidad Nacional Autónoma de México San Andrés Tuxtla Mexico; ^4^ Centro Interdisciplinario de Investigación para el Desarrollo Integral Regional Unidad Oaxaca Instituto Politécnico Nacional Santa Cruz Xoxocotlán Mexico

**Keywords:** arboreal camera trapping, biosphere reserve, canopy, habitat fragmentation, human‐modified landscape, species richness

## Abstract

Habitat loss threatens biodiversity worldwide being particularly detrimental in tropical rainforests where a cumulative deforestation expands for decades. Tropical rainforests harbor a rich mammal diversity with a wide range of species using different habitats, ranging from forest‐dwelling to arboreal species. Recent techniques such as camera trapping have proven to be useful to study the ecology of arboreal mammals. Here, we assessed the overall community structure of arboreal mammals in a protected area by analyzing the patterns of diversity and abundance and their spatial and seasonal variations. A total of 21 camera‐trapping stations were set in clusters in three zones. Spatial and seasonal alpha‐diversity and community evenness patterns were estimated using Hill's numbers, and Sørensen's dissimilarities were used as a proxy to estimate beta‐diversity. A relative abundance index was calculated for each species, at each site and season. To estimate the influence of spatial and tree morphology on arboreal use by mammals, a Principal Component Analysis was performed. We observed a high species richness (14 species) of arboreal mammals. Species richness remained similar between sites, although shifts in abundances and a decreasing gradient in community evenness related to the distance of camera trap station located in each site were noted. We observed a high‐ and low‐diversity dissimilarity between camera trap stations and between zones, respectively. Seasonality showed no significant effect over abundance, alpha, and beta diversities. This protected area holds the natural habitat conditions to ensure the persistence of this rich arboreal mammal community.

## Graphical abstract

Arboreal camera trapping provides insights about mammals' community in a human‐modified landscape. High species richness in the area nourish the notion of HML as refuges for fauna.

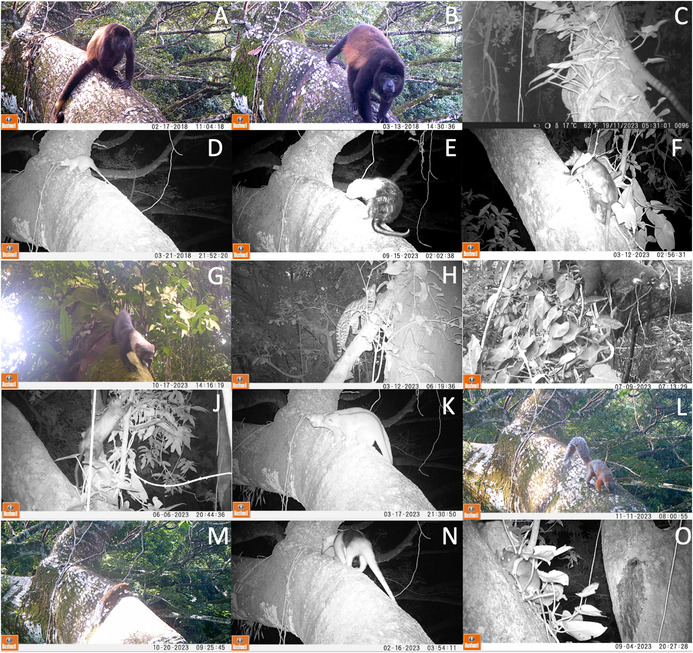

## 1 Introduction

Neotropical rainforests hold a rich mammal community including approximately 1700 species (Túnez et al. 2021). However, a rampant habitat loss over the last decades, illegal hunting of wildlife, and introduction of exotic species threaten its conservation (Túnez et al. 2021; Jaureguiberry et al. 2022). A direct consequence of human‐induced habitat loss is the fragmentation of large portions of pristine habitats into smaller fragments, subject to different disturbances. These reduced and fragmented habitats referred to as human‐modified landscapes (HML) can potentially shelter a high biodiversity under certain conditions (Wright and Muller‐Landau 2006; Melo et al. 2013; Arroyo‐Rodríguez et al. 2020). Nonetheless, identifying what these conditions are and how they affect biodiversity remain challenging and depend on the context of each HML (Chazdon et al. 2009; Melo et al. 2013). The impact of anthropic disturbances on Neotropical forests has been studied for both volant (Arroyo‐Rodríguez et al. 2016) and nonvolant mammals (Regolin et al. 2020). However, there are few studies quantifying the impact of anthropic disturbance on arboreal mammals, likely due to the challenges imposed to access the canopy (Anderson et al. 2015).

Rainforest canopies are considered habitat reservoirs of biodiversity worldwide due to their structural complexity and the diversity of microhabitats related to different environmental conditions along a height gradient (Seidler et al. 2013; Vinod et al. 2023). Biodiversity responds to such vertical stratification through a differential species assemblage (Moffett 2013), a phenomenon that has been studied on bats (Kennedy, Sillett, and Szewczak 2014), rodents (Taylor and Lowman 1996), and medium‐sized mammals (Agostini et al. 2022). Recently, the use of camera traps with climbing techniques has provided a methodological approach to study species of arboreal mammals (Anderson et al. 2015; Lowman 2021; Moore et al. 2021) from different perspectives, such as community ecology (Haysom et al. 2021; Arévalo‐Sandi et al. 2021; Agostini et al. 2022), species biotic interactions (Monteza‐Moreno et al. 2022; Marie et al. 2022), species responses to environmental variables (Nekaris, Handby, and Campera 2021), and the impact of habitat loss and fragmentation (Gregory et al. 2014; Whitworth et al. 2019; Cudney‐Valenzuela et al. 2023). Medium‐to‐large sized species of mammals living in canopies usually show morphological specialization, high energetic demands (Hanna and Schmitt 2011), a long lifespan (Oli and Dobson 2003), and relatively large dispersal ranges (Sukma et al. 2019; Broekman et al. 2023). Thus, these mammals are dependent on a high habitat quality (Cudney‐Valenzuela et al. 2023) and are particularly vulnerable to the impacts of human‐induced habitat disturbances (Almeida‐Rocha, Peres, and Oliveira 2017; Whitworth et al. 2019).

Camera‐trapping surveys on forest‐dwelling mammals have provided valuable insights on the impacts of human disturbances on medium‐sized species of mammals. For example, larger species of forest‐dwelling mammal communities decrease their abundances as a function of the closeness to human settlements, while smaller species tend to be more associated with anthropized sites (Lhoest et al. 2020; Li et al. 2022). Mammal species richness show contrasting results. For example, Lhoest et al. (2020) report a decrease in abundances from conserved to less conserved habitats. On the other hand, Li et al. (2022) found a major number of species related with an increment of human settlements and disturbed habitats. Furthermore, arboreal camera trapping has shown high correlations between species richness and abundance with old‐growth forests in fragmented landscapes (Cudney‐Valenzuela et al. 2023).

Recent studies using arboreal camera trapping have been conducted in tropical rainforests in Mexico. For example, Astiazarán‐Azcarraga, Gallina Tessaro, and Delfin‐Alfonso (2020) estimated the abundance and activity patterns of arboreal mammals in a protected area, and Pelayo‐Martínez et al. (2023) determined the activity patterns of arboreal mammals, respectively, in southeast Mexico. Hidalgo‐Mihart et al. (2022) quantified the use of artificial canopy bridges by arboreal mammals as a strategy to mitigate the highway fragmentation impacts on the species of mammals. Further, Cudney‐Valenzuela et al. (2021, 2023) evaluated the impact of anthropic disturbances on arboreal mammals in a fragmented landscape and determined the scale effects in which environmental variables influence arboreal mammals (Cudney‐Valenzuela et al. 2022). However, as the former was developed in a multipatch framework, the evaluation of arboreal mammal communities in a continuum corridor at a local scale remains necessary to complement the previous insights.

Here, we conducted an arboreal camera‐trapping survey in a tropical rainforest located at the Los Tuxtlas biosphere reserve (LTBR) in southeast Mexico. Los Tuxtlas Tropical Biology Station (LTTBS) is located in a highly fragmented landscape of tropical rainforest surrounded by areas devoted for agriculture and livestock (Coates 2017; Von Thaden et al. 2020). Due to the high mammal diversity described in the LTBR in which 52 species have been reported historically present (González‐Christen and Coates 2019), an update of the current mammal inventory might provide insights about the conservation status of this group.

In a previous study, a rich medium‐to‐large forest‐dwelling mammal community using camera‐trapping surveys was determined (Flores‐Martínez et al. 2014, 2022). In the present one, specifically, we aimed to (i) determine the species composition of arboreal mammals, their overall diversity, and patterns of abundance, (ii) analyze the effects of habitat heterogeneity and seasonality on species richness, evenness, and abundance in order to explore their spatiotemporal variations, and (iii) assess if there is a relationship between the presence of arboreal mammals and local spatial and tree morphological features. We anticipated that arboreal mammals exhibit higher species richness in areas associated with a greater proportion of old‐growth forest (Cudney‐Valenzuela et al. 2023) and a decrease in abundance as a function of the closeness to human settlements (Lhoest et al. 2020; Li et al. 2022). Further, we anticipated that location of camera traps and changes in seasonal conditions will promote spatial and temporal disparities in the abundances of arboreal mammals (Nekaris, Handby, and Campera 2021; Cosby et al. 2023). Finally, focal branch morphological variation is expected to influence arboreal mammals attributable to their kinematic adaptations (Shapiro and Young 2010).

## 2 Methods

### 2.1 Study Site

**FIGURE 1:**
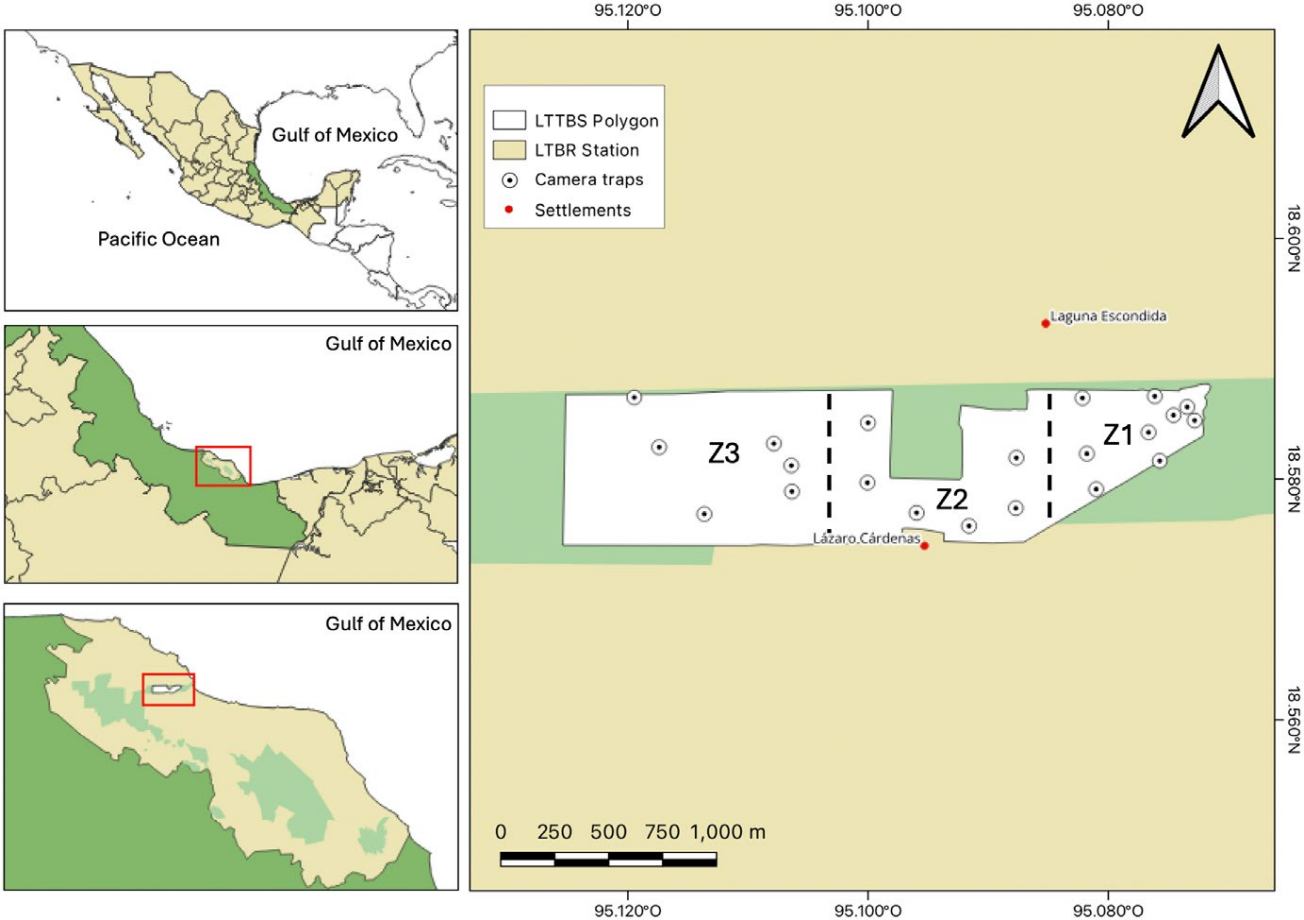
Geographical location of the Los Tuxtlas Tropical Biology Station in Mexico.

LTTBS is a protected area with 644 ha of undisturbed vegetation located in the State of Veracruz in southeast Mexico (95°04′ – 95°09′ W and 18°34′ – 18°36′ N) within the Los Tuxtlas Biosphere Reserve (LTBR; Figure 1) (Coates 2017). The area presents humid to subhumid tropical climate, with an annual mean temperature of 25°C and a rainy season from August to February (Soto and Gama 1997). The mean annual precipitation varies from 1500 to 4000 mm depending on the altitude (Lozano‐García et al. 2017). The vegetation in the LTTBS is a tropical rainforest with trees ranging from 30 to 35 m, which can decrease in areas with slopes (Cornejo‐Tenorio, Ibarra‐Manríquez, and Sinaca‐Colín 2019). The LTTBS is a rectangular‐shaped polygon surrounded by grasslands for livestock (Coates 2017; Von Thaden et al. 2020) (Figure 1).

### 2.2 Data Collection

We set a total of 21 camera traps in the canopy of the LTTBS since August 2022 to December 2023 (9 Bushnell Trophy Trail Camera HD119717, 8 UOVision Green 30, 3 Mixmart HC801, and 1 LTL Acorn 6210; Figure 1). The distance between cameras was ca. 638 m (SD = 316.93). Selection of trees was based on three criteria: (1) the geographic location of a pre‐established point grid to systematically cover the LTTBS area, (2) the identification of canopy bridges or foraging branches which arboreal mammals could use (Gregory et al. 2017), and (3) the absence of sites susceptible to the influence of nontarget stimuli (i.e., backgrounds with a high density of mobile branches and leaves) and potential risks (i.e., dead or weak branches or insect colonies) that may threaten the integrity of the climbers (Gregory et al. 2017; Moore et al. 2021).

A set of spatial variables such as the distance to the border, LTTBS buildings, human settlements, and water bodies were measured in Google Earth Pro (Google Earth Pro 2024). Human settlements were mainly associated with agricultural and hunting activities. The camera height (range = 6–20.9 m, mean = 13.80 m, SD = 4.53 m), focal branch diameter (range = 0.66–2.87 m, mean = 1.60, SD = 0.67 m), focal branch angle (range = 2°–76°, mean = 32.73°, SD = 23.86°), and DBH at 1.5 m (range = 1.7–5.5 m, mean = 3.04 m, SD = 1.30 m) were taken in situ and considered as tree‐morphological variables.

### 2.3 Species Identification

Mammalian species were identified with the comparison of the photo events with the handbook of mammal species of the LTTBS (Coates‐Estrada and Estrada 1986) and the databases of previous camera‐trapping surveys in the area (Flores‐Martínez et al. 2014, 2022).

### 2.4 Zone Selection

Camera traps were clustered into three zones (Figure 1) based on their closeness and the variation of four physical variables, as follows: Camera traps 1–9 were grouped in Zone 1 (Z1) nearby the LTTBS facilities; camera traps 10–15 in Zone 2 (Z2) close to the Lazaro Cardenas community, and camera traps 16–21 in Zone 3 (Z3), which is closely connected with the main patch of original cover of the volcano San Martin Tuxtla. The differences in environmental variables between sites (e.g., altitude, distance to border, distance to human settlements, and forest cover at 300 m buffers) were validated with a t test in RStudio (Figure S1; R Core Team 2022).

### 2.5 Data Analyses

A first species‐specific Relative Abundance Index (RAI) was obtained to compare with a previous ground‐based camera‐trapping survey at the LTTBS by multiplying the independent records (24‐h exclusion criterion) per 100 and dividing between the sampling effort (Flores‐Martínez et al. 2022). A second RAI was obtained only to compare with another arboreal camera trap survey performed in Veracruz (Astiazarán‐Azcarraga, Gallina Tessaro, and Delfin‐Alfonso 2020), by multiplying the independent records (1‐h exclusion criterion) per 1000 and dividing between the sampling effort. Rank‐abundance graphs were produced by splitting the records of arboreal mammals into their respective zones and plot differences between zones and seasons (rainy and dry) to visualize the temporal changes in mammal abundance. A paired *t*‐test was performed in RStudio to test differences in the first RAI values of species between seasons; mammal abundances were squared‐root‐transformed to meet the normality and homoscedasticity assumptions.

Alpha diversity analysis was performed using Hill's numbers with the iNEXT package in RStudio (Hsieh, Ma, and Chao 2016; R Core Team 2022). Diversity order values were obtained at the reference sample sizes, and an evenness factor (EF) was calculated as a proxy of the community abundance homogeneity. EF was obtained by dividing the diversity order *q*2 (i.e., dominant species) by diversity order *q*0 (i.e., species richness). Diversity accumulation curves were plotted through an extrapolation of the diversity order values to double the individual reference sample size (Chao and Jost 2012; Chao et al. 2014). The last process was repeated to assess variations in diversity values between seasons. Beta diversity analysis was assessed as Sørensen's dissimilarity index (β_SOR_) and their components (β_SIM_ = species turnover and β_NES_ = species nestedness) using presence/absence matrices with the betapart package in RStudio (Baselga and Orme 2012; Baselga et al. 2023; R Core Team 2022). Spatial beta diversity was assessed with the function beta.multi at an intra‐site level (i.e., diversity dissimilarities between camera‐trapping stations at the three sites) and at a site level (i.e., diversity dissimilarities between sites). Seasonal beta diversity analysis was conducted with the function beta.temp between matched site matrices.

Finally, a Principal Component Analysis (PCA) was performed with the FactoMineR package in RStudio (Le et al. 2008) in order to identify the effects of the spatial (i.e., altitude, distances to border, LTTBS facilities, human settlements, and to water sources) and morphological variables (i.e., camera height, DBH, branch diameter, and branch angle) over the species.

## 3 Results

**FIGURE 2:**
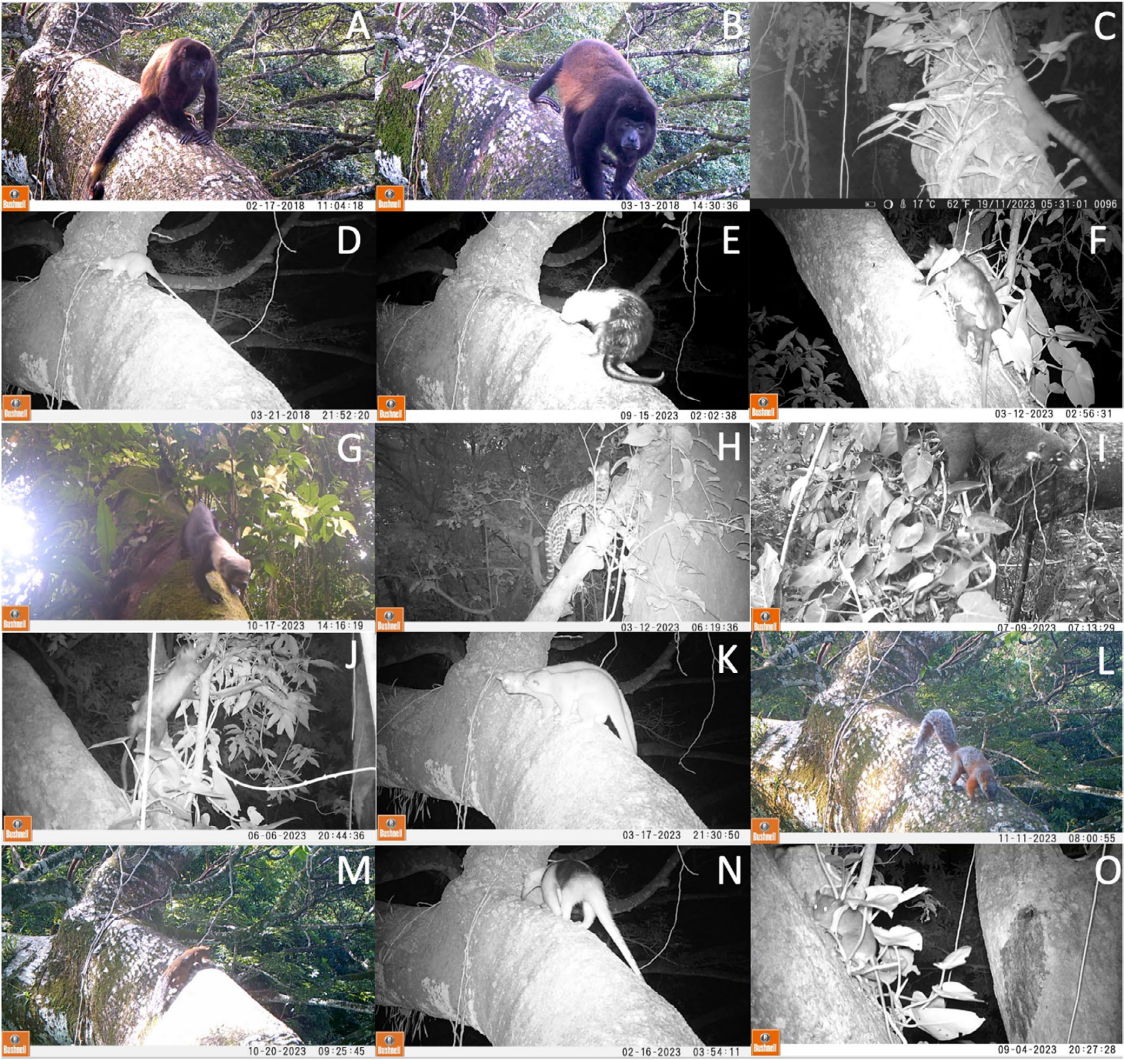
Pictures of the mammals registered in the canopy of the LTTBS. (A) Female individual of *A. palliata* , (B) Male individual of *A. palliata* , (C) *B. sumichrasti* , (D) *C. derbianus* , (E) *C. mexicanus* , (F) *D. marsupialis* , (G) *E. barbara* , (H) *L. wiedii* , (I) *N. narica* , (J) *P. opossum* , (K) *P. flavus* , (L) *S. aureogaster* , (M) *S. deppei* , (N) *T. mexicana* , and (O) *T. nudicaudus* .

The sampling effort was 4897 camera trap days in a 16‐month survey. Imperfect sampling efforts were caused by several factors such as battery and memory exhaustion caused by nontarget stimuli and camera malfunctioning due to environmental factors. A total of 2995 photograph events (records) were obtained, of which 2437 records contained species‐level identifiable medium‐sized mammals and 1212 effective records. Fourteen of the eighteen arboreal species of mammals reported in the LTTBS canopy were recorded in this study corresponding to five mammalian orders (Table 1; Figure 2). The clustering process of the 21 camera trap stations in the three zones was validated by the significative differences of 12 out of the 15 paired variables between areas, suggesting that overall, each site holds different environmental features (Figure S1).

**TABLE 1:** Species of mammals registered with arboreal camera trapping in the Los Tuxtlas Tropical Biology Station (LTTBS).

Species	Detections	Habit	RAI–24 h	Flores‐Martínez et al. 2022	RAI–1 h	Astiazarán‐Azcarraga, Gallina Tessaro, and Delfin‐Alfonso 2020	IUCN	NOM
*Alouatta palliata*	290	Arboreal	2.68	—	17.97	—	EN	P
*Bassariscus sumichrasti*	4	Semiarboreal	0.02	—	0.20	0.75	LC	Pr
*Caluromys derbianus*	95	Arboreal	0.98	—	16.54	—	LC	A
*Coendou mexicanus*	215	Arboreal	2.43	—	35.53	2.62	LC	A
*Didelphis marsupialis*	160	Semiarboreal	1.74	0.85	21.65	15.39	LC	—
*Eira barbara*	21	Semiarboreal	0.20	0.75	1.63	—	LC	P
*Leopardus wiedii*	4	Semiarboreal	0.06	2.24	0.82	2.25	LC	P
*Nasua narica*	491	Semiarboreal	2.66	13.57	32.67	3.00	LC	—
*Philander opossum*	2	Semiarboreal	0.02	—	0.20	0.37	LC	—
*Potos flavus*	458	Arboreal	5.54	—	72.08	9.38	LC	Pr
*Sciurus aureogaster*	142	Arboreal	2.11	—	20.83	19.89	LC	—
*Sciurus deppei*	226	Semiarboreal	1.68	4.38	27.98	—	LC	—
*Tamandua mexicana*	78	Semiarboreal	0.86	0.11	8.99	4.12	LC	P
*Tylomys nudicaudus*	251	Arboreal	2.56	—	30.84	7.50	LC	—

*Note:* The type of habit is mentioned; arboreal species are those that strictly live in the canopy, and semiarboreal are those which also inhabit the ground. The RAI indices of the species reported by Flores‐Martínez et al. 2022 and Astiazarán‐Azcarraga, Gallina Tessaro, and Delfin‐Alfonso 2020 were added to allow comparison. The conservation status of the species corresponds to the UICN Red List categories (IUCN, 2023): Endangered (EN), Near‐Threatened (NT), Least Concern (LC), and to the Mexican regulation norm categories (NOM‐059‐SEMARNAT‐2010): Endangered (P), Threatened (A), and Subject to Special Protection (Pr).

### 3.1 Species Abundance

**FIGURE 3:**
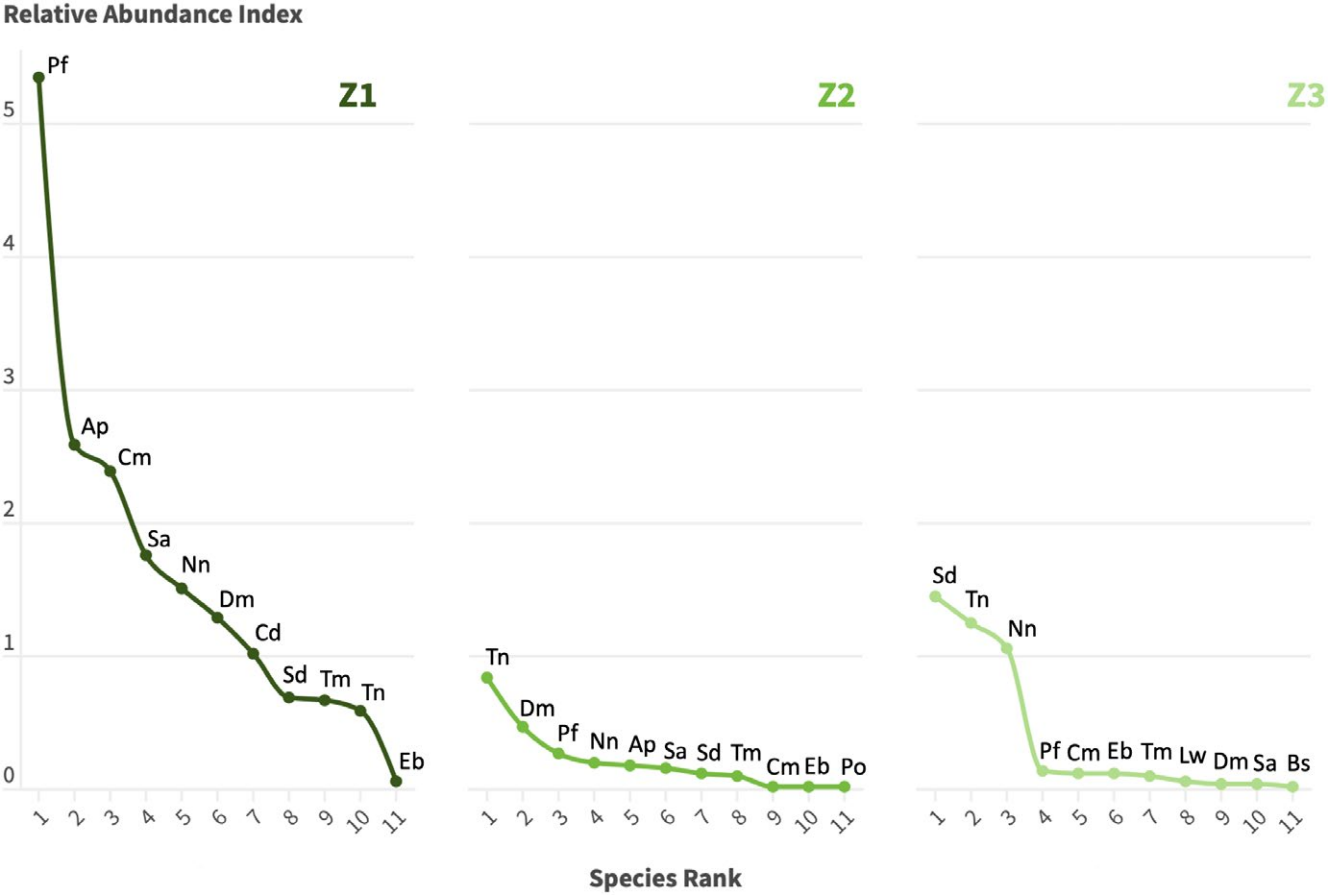
Rank‐abundance graph summarizing the differences in the abundances of the species between zones. Ap = *A. palliata* , Bs = *B. sumichrasti* , Cd = *C. derbianus* , Cm = *C. mexicanus* , Dm = *D. marsupialis* , Eb = *E. barbara* , Lw = *L. wiedii* , Nn = *N. narica* , Pf = *P. flavus* , Sa = *S. aureogaster* , Sd = *S. deppei* , Tm = *T. mexicana* , and Tn = *T. nudicaudus* .

*Potos flavus* was the most abundant species in the survey (overall RAI of 5.76), followed by *Alouatta palliata* and *Nasua narica* (RAI = 2.78 both). With a single effective record, *Bassariscus sumichrasti* and *Philander opossum* were the least abundant species (RAI = 0.02); *Leopardus wiedii* and *Eira barbara* also were species with low abundance indices (RAI = 0.06 and 0.20, respectively). Zone 1 showed the highest number of detections (*n* = 878), accounting for 11 species, and an overall RAI of 35.56. *P. flavus* and *A. palliata* were the most abundant species in this site (*n* = 262, RAI = 10.61 and *n* = 127, RAI = 5.14, respectively). *Tylomys nudicaudus* and *E. barbara* had the lowest number of detections (*n* = 29 and 3, respectively) and abundance values in this site (RAI = 1.17 and 0.12, respectively). *B. sumichrasti, L. wiedii*, and *P. opossum* were absent at Zone 1. Zone 2 had a total of 118 detections and an overall RAI of 9.70. This site had the same number of species (s = 11), where *T. nudicaudus* and *Didelphis marsupialis* were the most detected and abundant species (*n* = 41, RAI = 3.37, and *n* = 24, RAI = 1.97, respectively). *Coendou mexicanus, E. barbara*, and *P. opossum* were the least detected and abundant species (*n* = 1, RAI = 0.08 for both), and three species, *B. sumichrasti* , *Caluromys derbianus*, and *L. wiedii,* were absent at Zone 2. Overall, Zone 3 accounted for the second largest number of detections and abundance (*n* = 216, RAI = 17.84). A total of 11 species were recorded in this zone, in which *Sciurus deppei* and *T. nudicaudus* were the most detected and abundant species (*n* = 71, RAI = 5.86 and *n* = 61, RAI = 5.04). *B. sumichrasti* (*n* = 1, RAI = 0.08), *D. marsupialis*, and *Sciurus aureogaster* (*n* = 2, RAI = 0.17, for both) were the least detected species. *A. palliata* , *C. derbianus* , and *P. opossum* were not detected at this site (Table 2; Figure 3).

**TABLE 2:** RAI values of each mammalian species along the three zones assessed and between seasons.

Species	Rainy season				Dry season			
	SE = 2856 camera/days				SE = 2041 camera/days			
	Z1	Z2	Z3	Overall rainy	Z1	Z2	Z3	Overall dry
	SE = 1599 c/d	SE = 584 c/d	SE = 673 c/d		SE = 870 c/d	SE = 633 c/d	SE = 538 c/d	
*Alouatta palliata*	5.88	0.17	—	3.33	3.79	1.26	—	2.01
*Bassariscus sumichrasti*	—	—	0.15	0.04	—	—	—	—
*Caluromys derbianus*	2.69	—	—	1.51	0.80	—	—	0.34
*Coendou mexicanus*	4.38	—	0.59	2.59	5.40	0.16	0.37	2.45
*Didelphis marsupialis*	3.38	0.68	0.15	2.07	1.03	3.00	0.19	1.42
*Eira barbara*	0.19	—	0.74	0.28	—	0.16	0.19	0.10
*Leopardus wiedii*	—	—	—	—	—	—	0.56	0.15
*Nasua narica*	2.63	0.86	3.12	2.38	3.68	0.79	5.76	3.33
*Philander opossum*	—	—	—	—	—	0.15	—	0.05
*Potos flavus*	10.51	1.54	0.74	6.37	10.80	0.63	0.37	4.90
*Sciurus aureogaster*	3.25	1.03	—	2.03	3.91	0.32	0.37	1.86
*Sciurus deppei*	1.38	0.51	5.94	2.28	1.38	0.47	5.76	2.25
*Tamandua mexicana*	1.19	0.34	0.45	0.84	1.61	0.47	0.37	0.93
*Tylomys nudicaudus*	1.31	3.08	4.90	2.52	0.92	3.63	5.20	2.89
	36.76	8.22	16.79	26.23	33.33	11.06	19.14	22.68

Abbreviation: SE = sampling effort.

The number of effective records and RAI showed seasonal variations (Table 2; Figure S2). During the rainy season, 749 records were recorded with a sampling effort of 2856 camera trap days (RAI = 26.23). *P. flavus* was the most abundant species (*n* = 182, RAI = 6.37), followed by *A. palliata* (*n* = 95, RAI = 3.33), *C. mexicanus* (*n* = 74, RAI = 2.59), and *T. nudicaudus* (*n* = 72, RAI = 2.52). *E. barbara* (*n* = 8, RAI = 0.28) and *B. sumichrasti* (*n* = 1, RAI = 0.04) were the least abundant species, and no records of *L. wiedii* and *P. opossum* were recorded during the rainy season. On the other hand, during the dry season, a total of 463 records were obtained with a sampling effort of 2041 camera trap days (RAI = 22.68). *P. flavus* was the most abundant species (*n* = 100, RAI = 4.90), followed by *N. narica* (*n* = 68, RAI = 3.33), *T. nudicaudus* (*n* = 59, RAI = 2.89), and *C. mexicanus* (*n* = 50, RAI = 2.45). *L. wiedii* (*n* = 3, RAI = 0.15) and *P. opossum* (*n* = 1, RAI = 0.05) were detected only in the dry season, and *B. sumichrasti* was not recorded. No significative differences were observed in the abundance of arboreal mammals between seasons (*t* = −2.001, *p* = 0.0731). Further, Zone 1 showed the highest RAI values in both seasons between the three sites (wet season RAI = 36.76; dry season RAI = 33.33), followed by Zone 3 (wet season RAI = 16.79; dry season RAI = 19.14) and Zone 2 (wet season RAI = 8.22; dry season RAI = 11.06). Zone 1 showed a higher RAI in the rainy season; Zones 2 and 3 showed a higher RAI in the dry season (Table 2).

### 3.2 Species Diversities

All three sites showed the same *q*0 values (11 effective number of species). However, *q*1 and *q*2 orders showed differences; Zone 1 showed higher (7.99 and 6.37) effective common and dominant species than Zone 2 (6.84 and 5.18) and Zone 3 (5.12 and 4.01), respectively. Community evenness showed a decreasing pattern; Zone 1 showed the highest evenness factor (EF = 0.579) than Zone 2 (EF = 0.470) and Zone 3 (EF = 0.364), respectively. Zone 3 resulted as the most dominated community (Table 3; Figure S3). Further, for the seasonal diversity analyses, sample coverage was ≥ 0.99, denoting a high sample completeness. The values of each diversity order were similar for both seasons, starting at 12 effective species for *q*0 order at the rainy season and 13 effective species for the dry season. For the common species, the rainy season had a higher value (*q*1 = 9.20) than the dry season (*q*1 = 9.04), reaching an identical dominant species value (*q*2 = 7.90). Community evenness was similar with an evenness factor of 0.658 and 0.607 for the rainy and dry seasons, respectively (Table 3; Figure S4).

**TABLE 3:** Alpha diversity values along the three zones assessed in the LTTBS and at each season.

Zone	*N*	Sample coverage	*q*0	*q*1	*q*2	Evennes factor
			Species richness	Common species	Dominant species	
Z1	878	1.000	11.00	7.99	6.37	0.579
Z2	118	0.983	11.00	6.84	5.18	0.470
Z3	216	0.995	11.00	5.12	4.01	0.364
Rainy season	749	1.000	12.00	9.20	7.90	0.658
Dry season	463	0.997	13.00	9.04	7.90	0.607

*Note: N* = the total number of effective records for each zone, *q*0 = species richness, *q*1 = effective number of common species, *q*2 = effective number of dominant species.

Diversity dissimilarities between the sites showed similar results. Zone 1 (i.e., C1–C9) had a total dissimilarity of 0.701, where species turnover was 59.9% (*β*_SIM_ = 0.420) and species nestedness was 40.1% (*β*_NES_ = 0.281) of such dissimilarity. Zone 2 (i.e., C10–C15) showed the highest total dissimilarity among the three zones (β_SOR_ = 0.784), where species turnover was 69% (*β*_NES_ = 0.541) and species nestedness was 31% (*β*_NES_ = 0.242) of such dissimilarity. Zone 3 (i.e., C16–C21) had a total dissimilarity of 0.716, where species turnover was 62% (*β*_SIM_ = 0.444) and species nestedness was 38% (*β*_NES_ = 0.272). The dissimilarity between sites was low (*β*_SOR_ = 0.208) represented completely by species turnover (*β*_SIM_ = 0.136). Seasonal dissimilarities also showed low values at the sites, where Zone 1 (*β*_SOR_ = 0.047) and Zone 2 (*β*_SOR_ = 0.157) differences between seasons resulted from nestedness. Conversely, for Zone 3 (*β*_SOR_ = 0.157), species turnover represented the highest proportion of the dissimilarity (*β*_SIM_ = 0.111, *β*_NES_ = 0.046).

### 3.3 Principal Component Analysis

The first three dimensions of the PCA showed a total explained variance of 92.22%. Dimension 1 (dim1) accounted for 58.73% of such variance, on which both spatial and morphological variables showed similar contributions (Figure S5). Pairs of variables such as the branch angle, distance to border (*R* ^2^ = 0.91 and 0.94, respectively; *p* < 0.05), as well as altitude and distance to the LTTBS (*R* ^2^ = 0.91 and 0.96, respectively, *p* < 0.05), and camera height solely (*R* ^2^ = −0.90, *p* < 0.05) were the variables highly associated with dim1 (Figure S5). A group consisting of five of the seven strictly arboreal species ( *A. palliata* , *C. derbianus* , *P. flavus* , *S. aureogaster* , and *T. mexicana)* were positively associated with camera height and negatively with the pairs of branch angle–distance to border, and altitude‐distance to the LTTBS (Figure S5). *B. sumichrasti* , *S. deppei* , and *T. nudicaudus* were mainly associated with dim1. The pair of altitude and distance to the LTTBS was positively related with *S. deppei* in synergy with the branch diameter, the last variable being more associated with *B. sumichrasti*. Further, *T. nudicaudus* was clearly positively related to the pair of branch angle–distance to border, while *L. wiedii* was the most influenced species by dim1 highly positively associated with the branch diameter and altitude–distance to the LTTBS. In the case of *D. marsupialis,* a negative association was observed with the increase in the distance to human settlements and the DBH (Figure S5). Dimension 2 (dim2) explained 23.4% of the total variance (82.13% cumulative variance), the distance to water sources (*R* ^2^ = 0.91, *p* < 0.05) and DBH (*R* ^2^ = 0.83, *p* < 0.05) being the only significatively correlated variables with this dimension (Figure S5). Thus, *P. opossum* was the most associated species with dim2 showing a negative influence with the increase of the distance to water sources (Figure S5).

## 4 Discussion

### 4.1 Species Diversity

We were able to detect more than two‐thirds of the arboreal mammal species reported for the LTTBS. Some species which are reported in the area were not detected by our camera‐trapping protocol. For example, the lack of detections of spider monkey *Ateles geoffroyi* is consistent with the absence of sights of this species in over 30 years in the LTTBS. Thus, one reason may be that this species requires larger extensions of forest due to their large home ranges, and probably it is restricted to more inaccessible areas associated with the surrounding of the San Martín volcano (McLean et al. 2016) (Figure 1). Another nondetected species was the ocelot *Leopardus pardalis*. Interestingly, this species has not been recorded as an arboreal species in different locations of tropical rainforests in Mexico (Astiazarán‐Azcarraga, Gallina Tessaro, and Delfin‐Alfonso 2020; Cudney‐Valenzuela et al. 2021; Hidalgo‐Mihart et al. 2022; Pelayo‐Martínez et al. 2023), nor in many other neotropical areas (Gregory et al. 2014; Withwort et al. 2016; Bowler et al. 2017; Haysom et al. 2021), except for a single report in southeast Peru (Whitworth et al. 2019). Ocelots cannot be disregarded as semi‐arboreal species due to their capacity to climb trees (Murray and Gardner 1997). *Marmosa mexicana,* even if not detected in our arboreal camera‐trapping method, is expected to occur in the LTTBS due to the collected individuals deposited in the Mammals National Collection of the Biology Institute, UNAM. Further, *Cyclopes dorsalis* has not been confirmed yet to occur at the LTTBS. However, its potential distribution includes this area, and it is anticipated to occur (Coates‐Estrada and Estrada 1986). We expect to record this species by increasing the arboreal camera‐trapping sampling effort at the LTTBS.

The species richness obtained in our study (14 species) showed contrasting values with other studies conducted in tropical rainforests in Mexico. On the one hand, our study showed similar values with Astiazarán‐Azcarraga, Gallina Tessaro, and Delfin‐Alfonso (2020) that reported 12 species in another locality of Veracruz, and Cudney‐Valenzuela et al. (2021) reported 15 species in Marqués de Comillas in Chiapas, Mexico. Other studies reported a lower number of arboreal species of mammals in other tropical rainforest landscapes, with Pelayo‐Martínez et al. (2023) reporting six species and Hidalgo‐Mihart et al. (2022) reporting four species. Further, we observed that seven out of nine species of arboreal mammals shared with the study of Astiazarán‐Azcarraga, Gallina Tessaro, and Delfin‐Alfonso (2020) showed higher RAI values at LTTBS (Table 1). The above results show that the LTTBS holds a high species richness of arboreal mammals despite its history of habitat disturbance and still has the capacity to shelter a high number of native species in the canopy. Further, the high species richness of arboreal mammals reported in our study suggests that our sampling protocol was effective in recording even some elusive species (Moore et al. 2021). Moreover, our study surveying arboreal species of mammals can be complemented with previous studies conducted in the same sites monitoring forest‐dwelling medium‐sized mammals (Flores‐Martínez et al. 2022). Thus, it is important to design inventory studies complementing sampling protocols involving camera trap surveys of arboreal and ground‐dwelling species (Agostini et al. 2022). For example, we have currently reported 25 species of medium‐sized mammals (see Flores‐Martínez et al. 2014, 2022; Ríos‐Solís et al. 2021; this study) of the 31 species of mammals documented almost 40 years ago at the LTTBS (Coates‐Estrada and Estrada 1986).

### 4.2 Spatial Species Richness

The equality of species richness values between the three sites was somewhat unexpected given the location of sites. For example, we anticipated a lower species richness of arboreal mammals near human settlements, such as the Lázaro Cárdenas community (Zone 2), compared to the other zone located (Zone 3) in larger extensions of forests at the LTTBS. These results coincide with those of Li et al. (2022), where these authors found a similar species richness in different types of fragments. Conversely, Lhoest et al. (2020) found a decreasing trend in the species richness of mammals related to the presence of human settlements. Further, Cudney‐Valenzuela et al. (2023) observed a higher species richness in larger than smaller fragments of tropical rainforests. Our study contrasts with the latter two studies where declines in species richness of arboreal mammals were associated to human settlements and smaller habitat fragments. It is likely that the differences result from the fact that our three sites were mostly connected along a continuous corridor of tropical rainforest at LTTBS (Figure 1). Furthermore, our study included sampling sites at a local scale that the observed trends do not necessarily reflect larger‐scale landscape matrices (Riva and Fahrig 2023). The last could suggest that even if environmental differences are found in the area, the distance is no larger enough to influence this ecological metric. Thus, it is important to expand our study sites to a larger‐scale landscape framework at the LTBR. Interestingly, our study documented more species of arboreal mammals compared with the previous studies conducted at LTTBS, where methods were restricted to observing a lower herbivory, direct sightings, and tracks of medium‐sized mammals. Thus, it is likely that this discrepancy is due to the different methods used, rather than a result of a defaunation due to habitat loss and fragmentation (Dirzo and Miranda 1990; Mendoza 2005). Thus, it is important to establish a more integrated standard methodological protocol including camera trapping, direct observations, presence of tracks and scats, and rates of herbivory for documenting medium‐sized mammals in tropical forests (Silveira, Jácomo, and Diniz‐Filho 2003; Garden et al. 2007).

### 4.3 Species Abundances

Five of the seven strictly arboreal species recorded ( *A. palliata* , *C. derbianus* , *C. mexicanus* , *P. flavus* , and *S. aureogaster* ), and *T. mexicana* , showed their highest abundances in the Zone 1 (Table 2). The high association of variables, such as distance to the LTTBS and altitude with these species, is related to the location of this zone within the study site. The last due to the cameras of this zone were located near the facilities of the LTTBS, which also are the most distant from the San Martin Tuxtla volcano highlands. The increase in distance to the border showed a negative relationship with the same group of species (Figure S5), suggesting these mammals tend to respond positively to areas near borders. This coincides with the reports of Estrada, Coates‐Estrada, and Vazquez‐Yanes (1984) at LTTBS, who describe an increase in the growing and fruit production of *Cecropia obtusifolia* promoting a food source that allows high abundances of arboreal and ground‐dwelling mammals in the proximity of man‐made clearings.

The high abundance of *P. flavus* is consistent with three of the previous arboreal camera‐trapping surveys performed in Mexico, where *P. flavus* ranked the top three most abundant species (Astiazarán‐Azcarraga, Gallina Tessaro, and Delfin‐Alfonso 2020; Cudney‐Valenzuela et al. 2021; Pelayo‐Martínez et al. 2023). The high abundance of this species in Zone 1 could be partly explained by a temporal segregation with *N. narica*, which has been suggested to be a behavior of procyonids to avoid predators (Gómez‐Ortiz, Monroy‐Vilchis, and Castro‐Arellano 2019). In this case, due to the lack of an obvious top predator, rather than an antipredation strategy, the segregation between both species could be attributed to a niche partitioning strategy. Even if *P. flavus* seems to be highly favored by this temporal segregation based on its RAI, in the case of *N. narica,* the spatiotemporal overlap with *A. palliata* could be reflected in its lower abundance compared with the other procyonid. Competition between *N. narica* and *A. palliata* has been reported previously in the canopy of the LTTBS by ground‐based observations. Asensio, Arroyo‐Rodríguez, and Cristóbal‐Azkarate (2007) reported encounters between these species in which *A. palliata* commonly appears to exclude *N. narica* from trees providing food supply. The higher abundance of *A. palliata* coincides with the dominance of this species over *N. narica* (Table 1).

Further, the decrease in the abundances of *P. flavus, N. narica*, and *A. palliata* and the increase of the RAI of smaller species such as *T. nudicaudus* and *D. marsupialis* in Zone 2 may be related to the closeness of this area with the Lazaro Cardenas community. Distance to human settlements seems to be related to a decrease in community body‐size mean (Lhoest et al. 2020; Li et al. 2022), suggesting that this trend occurs in ground‐dwelling and arboreal mammals. Furthermore, *T. nudicaudus* was positively associated with both the increase of branch angle and distance to border (Figure S5). While the role of the focal branch characteristics over the species‐specific responses requires the incorporation of a kinematic focus, out of the scope of the present project, arboreal camera‐trapping proved to be a methodology that might extend in the future this kind of analysis to animals in their natural habitat (Cronin 2021). Moreover, the relation between this species and the increase in the distance to the border may be due to the fact that *T. nudicaudus* prefers sites far from human matrices to avoid competence with invasive rodents commonly associated with modified lands as *Rattus spp*. which have shown capabilities to also occupy the canopy (Nance et al. 2024). The merge of arboreal camera‐trapping and direct trapping methods which have been applied successfully in rodent surveys (Nance et al. 2024) may nourish the data obtained, allowing the recognition of the non‐identified species of this project and promoting more accurate assessments about the composition and dynamics of rodents communities in the canopy of the LTTBS.

The increase in abundance of *D. marsupialis* in Zone 2 differs from the results of Cruz‐Salazar et al. (2016), who report a decrease in the abundance of this species as a response to human disturbance. It is worth considering that in such a study, the two *Didelphis* species are present in the same site. In this case, the high abundance of this species in Zone 2 may be explained by its opportunistic and omnivorous behavior (Cordero and Nicolas 1987; Voss and Jansa 2021; Rojas‐Sánchez et al. 2023). This behavior and feeding habits likely allow this species to take advantage of human residues as a stable food resource, as seen for *Didelphis* (Cordero and Nicolas 1987; Glebskiy et al. 2024), which is a fact also supported by the association of *D. marsupialis* with the decrease in the distance to human settlements (Figure S5).

The high abundance of *S. deppei* in Zone 3 can be explained by the preference of this species to inhabit heavily dense and well‐covered areas (Best 1995), whereas *S. aureogaster* is reported to have higher plasticity to adapt to disturbed habitats (Koprowski et al. 2017), as in Zone 1 and 2 (Table 2). Thus, the negative effect of altitude on *S. aureogaster* is likely since Zone 3 is located in a more conserved area and at a higher altitude (Table 2), thus conversely, positively related to *S. deppei* (Figure S5).

Moreover, some species such as *A. palliata* and *S. aureogaster* exhibit tolerance to the human presence and commonly are associated to the sites near human settlements. However, a major association of these and the other strictly arboreal species with the nearness of the LTTBS was observed, rather than another kind of human settlement (Figure S5). Suggesting that even if both are sites with human presence and activity, there exists a positive effect of the LTTBS as a refuge for mammals (Flores‐Martínez et al. 2014, 2022).

It must be considered that even if some insights for *B. sumichrasti* , *L. wiedii* , and *P. opossum* were observed through analyses, the scarcity in their records (less than five detections) may lead to misinterpretations, for what the information reported about such species must be treated cautiously.

### 4.4 Species Vertical Use Preference

*A. palliata* , *C. derbianus* , *C. mexicanus* , *P. flavus* , and *S. aureogaster* showed a strong association with camera height, which may work as a proxy of the height at which animals occur. The last suggests that the occupancy of strictly arboreal species depends partly on the tree height, due to the relationship of this variable with the provision of food and shelter (Arroyo‐Rodríguez et al. 2007).

Further, from the six species that occurred in both the understory and the canopy of the LTTBS, *D. marsupialis* and *T. mexicana* showed higher RAI in the canopy than in the ground (Flores‐Martínez et al. 2022). Thus, the preference of *D. marsupialis* to occupy the canopy rather than the ground differs from observations reported in lowland forests in French Guiana; this opossum was recorded by direct captures more frequently on the ground in old‐grown forest than in the canopy (Adler et al. 2012). The preference to arboreality by *T. mexicana* also differs from previous reports in which this species is considered to inhabit mainly on the ground rather than in the canopy, which uses 40% of its active time (Navarrete and Ortega 2011). Even if it is worth considering that camera‐trapping surveys assessing the abundance of this species at both strata of the same site are scarce, *T. mexicana,* at least in LTTBS, may exhibit a more arboreal behavior based on its RAI and the similarity of its responses with strictly arboreal species observed in the PCA (Figure S5).

For *S. deppei* , its tendency to descend to the understory to forage could be a mechanism promoting its higher abundance in the ground, as reported by Flores‐Martínez et al. (2022) in comparison to our study (Coates‐Estrada and Estrada 1986; Best 1995). Also, we observed a higher RAI for *E. barbara* and *N. narica* in the ground (Flores‐Martínez et al. 2022), despite that other camera‐trapping studies have provided direct evidence of arboreal behavior for these species (Astiazarán‐Azcarraga, Gallina Tessaro, and Delfin‐Alfonso 2020; Cudney‐Valenzuela et al. 2023). There is clearly a need to conduct camera trap surveys in the canopy and in the ground simultaneously to assess their habitat preferences at different locations (Agostini et al. 2022).

### 4.5 Seasonal Species Abundances

We expected seasonal differences in the abundance of arboreal species of mammals given the phenological changes in the vegetation observed at the LTTBS (Estrada and Coates‐Estrada 1985; Cornejo‐Tenorio, Ibarra‐Manríquez, and Sinaca‐Colín 2019). Most arboreal mammals depend on fruit and seed production of trees to accomplish their metabolic requirements (Estrada, Coates‐Estrada, and Vazquez‐Yanes 1984; Coates‐Estrada and Estrada 1986). We observed a 38% decrease in the number of records of arboreal mammals from the rainy to the dry season (Table 2), although the difference was not statistically significant. We also observed similar values in species richness in both seasons (12 and 13 species respectively), and a low difference in community evenness recorded in the rainy than the dry season (Table 2), suggesting that communities conserve their general structure year‐round. It has been documented that most arboreal mammals show a nonmigratory behavior at the LTTBS (Coates‐Estrada and Estrada 1986). Species‐by‐species seasonal differences were observed in *B. sumichrasti, L. wiedii*, and *P. opossum* . *Bassariscus sumichrasti* occurred only in the rainy season; the latter two species occurred only in the dry season. We believe that more than the effect of seasonality, it is the cryptic behavior of certain arboreal species of mammals that can result in an absence of records. This is the case of *B. sumichrasti* (Pozos‐López, González‐Ruiz, and Ramírez‐Pulido 2024), the preference of *L. wiedii* to inhabit dense forest fragments (Pérez‐Irineo, Santos‐Moreno, and Hernández‐Sánchez 2017) or the preference of *P. opossum* to use the ground (Coates‐Estrada and Estrada 1986).

It is likely that the environmental changes associated with seasonality in our study area have little or no effect on the ecological metrics that we evaluated, compared to the strong seasonal differences observed in mammal communities in other highly dynamic environments such as flooded forests (Gonçalves et al. 2022). We also acknowledge some limitations of our study that could have biased some observed trends. For example, our study was limited to 1 year; a multi‐year analysis involving changes in community structure of arboreal mammals is needed to produce more reliable information. This is the case for *A. palliata* which has been extensively studied at LTBR (Dunn, Cristóbal‐Azkarate, and Veà 2010). Second, seasonality is known to influence the physiological responses of species and reproductive activity of arboreal mammals (Dubost and Henry 2017). Further, integrating more microclimate variables can help to better describe the fine‐scale environmental conditions of arboreal mammals (Fawcett et al. 2019; Vinod et al. 2023).

### 4.6 Community Evenness

We observed a decreasing trend in the community evenness related to the distance to the LTTBS facilities (Table 3). In a multisite ground‐based camera‐trapping analysis, Ahumada et al. (2011) reported that areas surrounded by human‐induced disturbed habitats were associated with more dominated mammal communities. In contrast, our results show that the closest site to disturbed habitat had the highest values of community evenness. Community evenness is commonly used as a proxy of disturbance and its decrease as a species extinction indicator (Rohr et al. 2016). The negative correlation between the distance to border and community evenness could be explained by the attraction of mammal species to the LTTBS human‐made forest edges due to the high abundance of permanent fruiting species such as *Cecropia obtusifolia* (Estrada, Coates‐Estrada, and Vazquez‐Yanes 1984). A second explanation may reside in the value of the patrols of forest guards near the facilities of the LTTBS. Similar gradients have been reported as a function of the type of area, where conservation‐intended sites present higher species richness values than community areas (Lhoest et al. 2020). Even if some metrics differ, the principle of the conservation value of protected areas in HML must be highlighted, suggesting that the LTTBS can be considered an important refuge for the arboreal mammal community (Bovo et al. 2018; Melo et al. 2013; Flores‐Martínez et al. 2022).

### 4.7 Communities' Dissimilarities

We found high species dissimilarities detected in the different sites. The higher proportion of species turnover can be interpreted that when we consider each camera trap as a community, species tend to be replaced rather than excluded. Beta analysis between zones showed a low dissimilarity in which due to the higher proportion of the species turnover component, the variation in the arboreal mammal communities is low at the LTTBS and mostly represented by a species reshuffle more than the loss of species. Further, all morphological variables proved to be significant within the PCA and poorly associated between them (Figure S5). For instance, based on the notion that in order to obtain resources, arboreal mammals use a wide range of movement strategies and exhibit different branch type preferences (Shapiro and Young 2010; Young et al. 2015; Youlatos et al. 2018; Wölfer et al. 2021), the focal branch characteristics are expected to influence the species recorded, on which branch angle (i.e., flat or vertical) seems to be more important for arboreal marsupials with similar kinematics than arboreal primates, than branch diameter (Shapiro and Young 2010). This coincides with the significative influence of camera height and focal branch angle over the strictly arboreal species detected in our study.

In sum, the LTTBS showed a high species richness and abundance of arboreal mammals compared to other localities showing larger fragments of tropical rainforests. These results highlight the importance of the LTTBS as a refuge of species of arboreal mammals and biodiversity despite of the high deforestation in the surrounding areas. The acquisition of more lands to the LTTBS could provide new secure shelter areas for their long‐term conservation.

## Author Contributions

**J. Vladimir Rojas‐Sánchez:** conceptualization (lead), data curation (lead), formal analysis (lead), funding acquisition (equal), investigation (lead), methodology (lead), project administration (equal), resources (equal), supervision (lead), validation (equal), writing – original draft (lead), writing – review and editing (lead). **Rosamond Ione Coates:** data curation (equal), investigation (equal), project administration (equal), resources (lead), supervision (equal), validation (lead), writing – original draft (supporting), writing – review and editing (lead). **Víctor Sánchez‐Cordero:** conceptualization (equal), formal analysis (equal), funding acquisition (lead), investigation (equal), project administration (supporting), resources (lead), validation (lead), writing – original draft (supporting), writing – review and editing (lead). **Mario C. Lavariega:** conceptualization (supporting), formal analysis (supporting), investigation (equal), methodology (equal), validation (equal), writing – original draft (equal), writing – review and editing (equal). **José J. Flores‐Martínez:** conceptualization (lead), data curation (lead), formal analysis (lead), funding acquisition (equal), investigation (lead), methodology (lead), project administration (equal), resources (equal), supervision (lead), validation (equal), writing – original draft (lead), writing – review and editing (lead).

## Conflicts of Interest

The authors declare no conflicts of interest.

## Target Audience

Conservation researchers.

## Supporting information


Data S1.

## Data Availability

The data and scripts supporting the analyses of the present study can be found in the following URL: https://bit.ly/4bXZ1sM.
