# Supplementary material for: Diversity and Abundance of the Species of Arboreal Mammals in a Tropical Rainforest in Southeast Mexico
Source: Ecol Evol. 2025 Jan 20;15(1):e70812. doi: 10.1002/ece3.70812 (PMC11746937; doi:10.1002/ece3.70812)
Supplement: Supplementary file 1 — Data S1. [file ECE3-15-e70812-s001.docx]

**Supporting Information**

Additional supporting information may be found in the online version of the article at the publisher’s website.

| Z1 - Z2 | Altitude | CI  LCI | UCI | Distance to border | CI  LCI | UCI | Distance to  LTTBS | LCI | CI  UCI | Distance to settlements | LCI | CI  UCI | Distance to water | LCI | CI  UCI |
| --- | --- | --- | --- | --- | --- | --- | --- | --- | --- | --- | --- | --- | --- | --- | --- |
|  | 0.8105 | -96.75 | 77.53 | 0.9172 | -190.71 | 173.06 | 0.0002* | -2306.3 | -1129.1 | 0.0197* | 102.05 | 909.42 | 0.0046* | 219.72 | 972.17 |
| Z1 - Z3 | 0.0029* | -285.46 | -73.08 | 0.0102* | -452.70 | -73.49 | 0.0002* | -2565.3 | -1065.8 | 0.5457 | -1300.1 | 17.01 | 0.0137* | 1368.8 | -209.32 |
| Z2 - Z3 | 0.0022* | -253.36 | -85.96 | 0.0047* | -410.60 | -97.90 | 5.56 e-8* | -4179.2 | -2887.4 | 0.0047* | -1832.0 | -462.57 | 0.0006* | -1966.4 | -803.5 |

Figure S1. Matrix summarizing the p-values of the t-student tests performed between the sets of data of each local variable at the three zones. *Significative values (i.e. p-value ≤ 0.05).

Section Methods, Lines 237-238, and Results, Line 294-297.


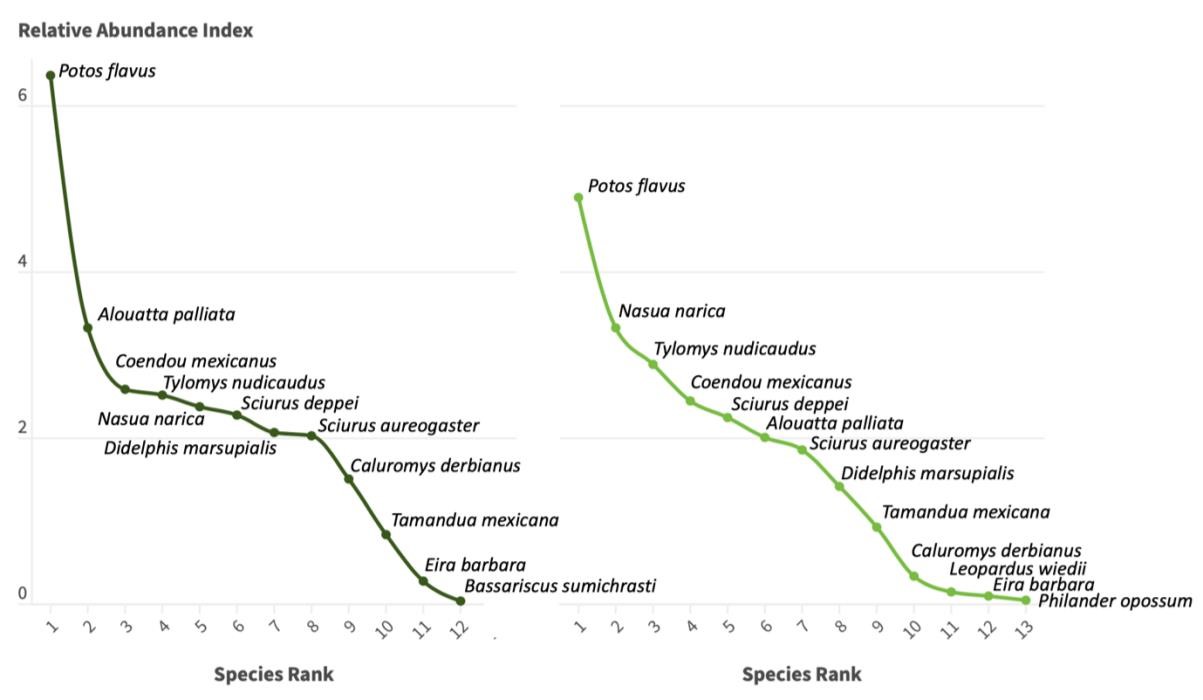


Figure S2. Rank-abundance graph summarizing the differences in the abundances of the species between seasons. Rainy season at left and dry season at right. Section Results, Lines 321-322.


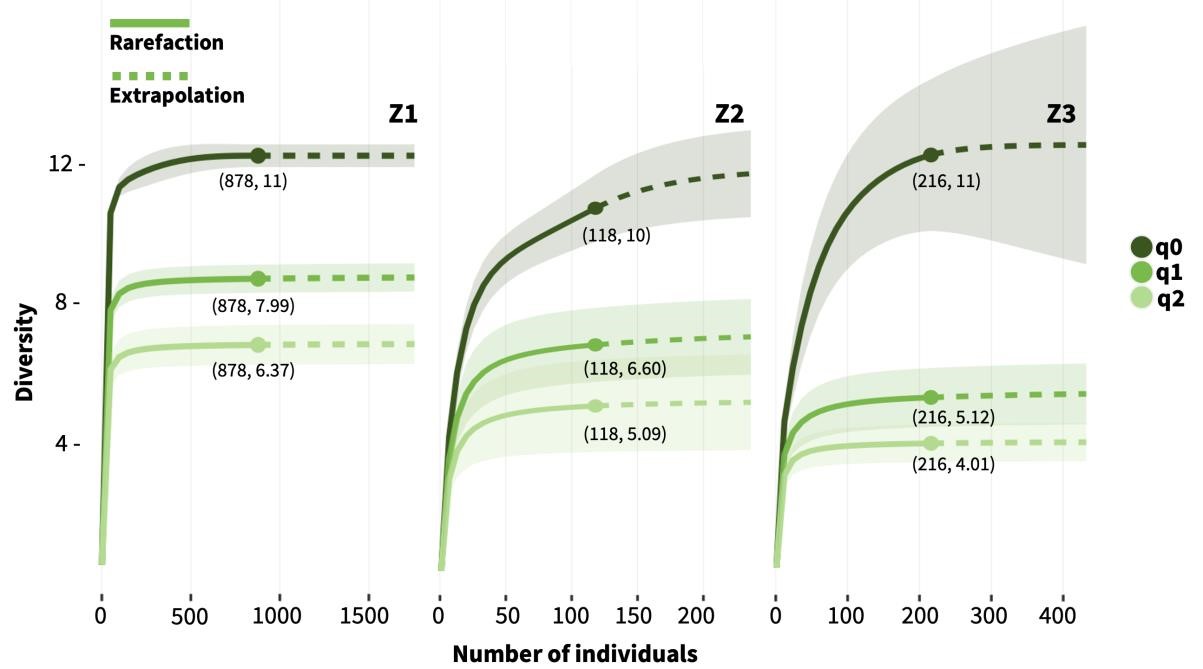


Figure S3. Diversity accumulation curves summarizing the three diversity orders values on each zone assessed. Section Results, Line 348.


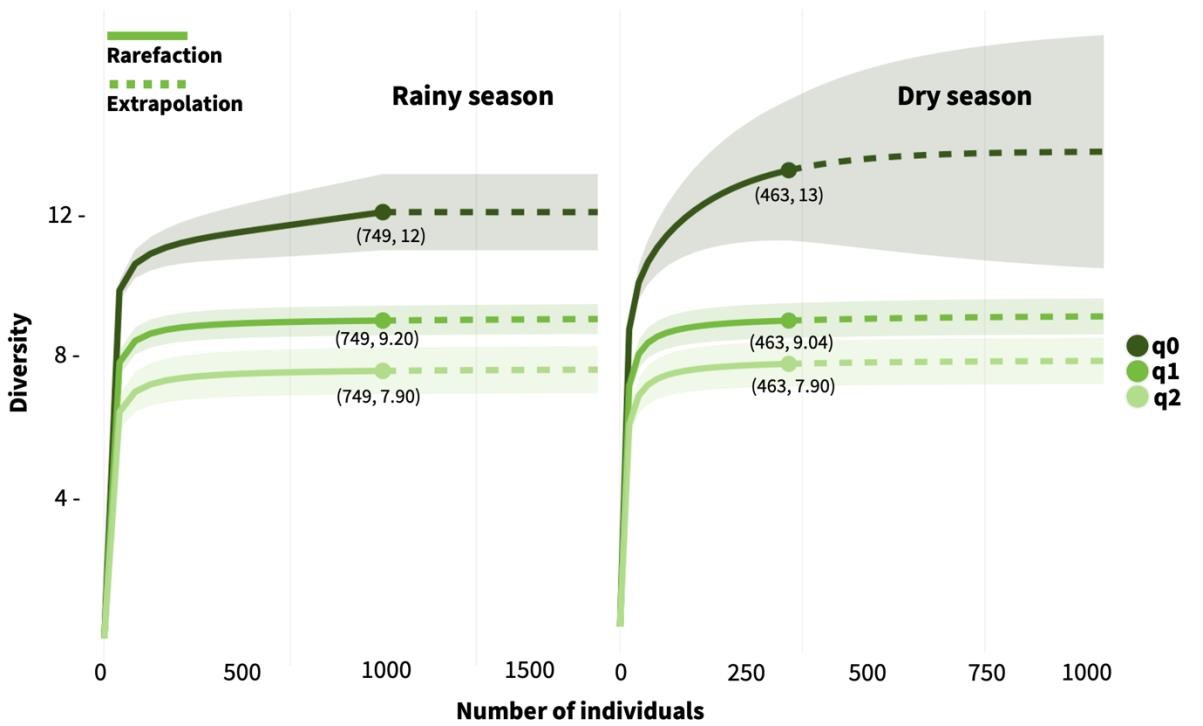


Figure S4. Diversity accumulation curves summarizing the diversity values of both the wet and the dry season. Section Results, Line 355.


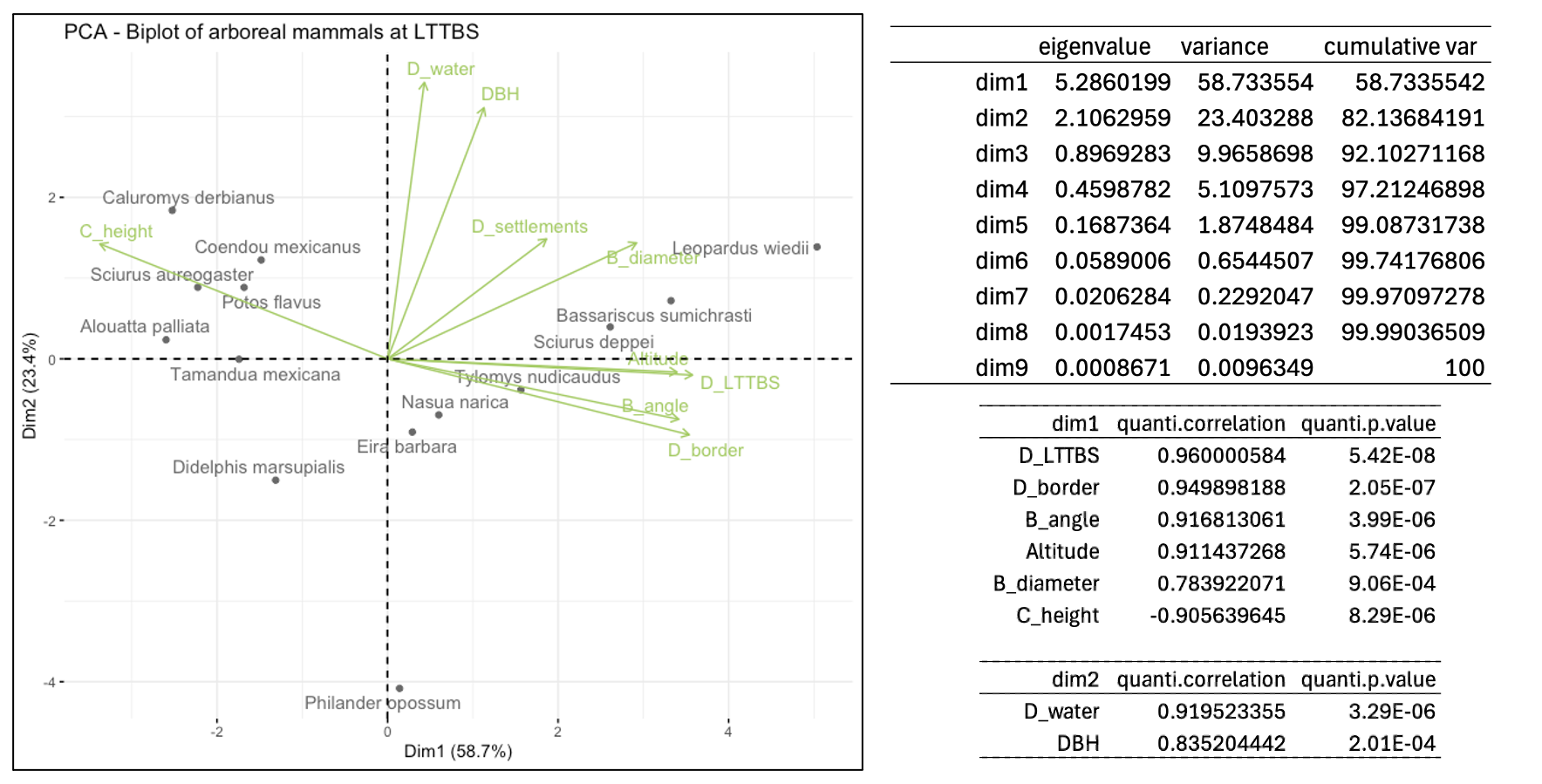


Figure S5. Principal Component Analysis (PCA) output information. Section Results, Line 393-414, and Discussion, Lines 493-495, 523-524, 542-545, 550-552, 554-556, 582-585, 648-649.
